# Early high-dose vitamin C in post-cardiac arrest syndrome (VITaCCA): study protocol for a randomized, double-blind, multi-center, placebo-controlled trial

**DOI:** 10.1186/s13063-021-05483-3

**Published:** 2021-08-18

**Authors:** Sander Rozemeijer, Harm-Jan de Grooth, Paul W. G. Elbers, Armand R. J. Girbes, Corstiaan A. den Uil, Eric A. Dubois, Evert-Jan Wils, Thijs C. D. Rettig, Arthur R. H. van Zanten, Roel Vink, Bas van den Bogaard, Rob J. Bosman, Heleen M. Oudemans-van Straaten, Angélique M. E. de Man

**Affiliations:** 1 Department of Intensive Care Medicine, Research VUmc Intensive Care (REVIVE), Amsterdam Cardiovascular Science (ACS), Amsterdam Infection and Immunity Institute (AI&II), Amsterdam Medical Data Science (AMDS), Amsterdam UMC, Location VUmc, Vrije Universiteit Amsterdam, De Boelelaan 1117, 1081 HV Amsterdam, The Netherlands; 2 Department of Anesthesiology, Amsterdam UMC, Location VUmc, Vrije Universiteit Amsterdam, De Boelelaan 1117, 1081 HV Amsterdam, The Netherlands; 3 Department of Intensive Care Medicine, Maasstad Hospital, Maasstadweg 21, 3079 DZ Rotterdam, The Netherlands; 4 Department of Cardiology, Erasmus University Medical Center, Dr. Molewaterplein 40, 3015 GD Rotterdam, The Netherlands; 5 Department of Intensive Care Medicine, Erasmus University Medical Center, Dr. Molewaterplein 40, 3015 GD Rotterdam, The Netherlands; 6 Department of Intensive Care Medicine, Franciscus Gasthuis & Vlietland, Kleiweg 500, 3045 PM Rotterdam, The Netherlands; 7 Department of Anesthesiology, Intensive Care and Pain Medicine, Amphia Hospital, Molengracht 21, 4818 CK Breda, The Netherlands; 8 Department of Intensive Care Medicine, Gelderse Vallei Hospital, Willy Brandtlaan 10, 6716 RP Ede, The Netherlands; 9 Division of Human Nutrition and Health, Wageningen University & Research, HELIX (Building 124), Stippeneng 4, 6708 WE Wageningen, The Netherlands; 10 Department of Intensive Care Medicine, Tergooi Hospital, Van Riebeeckweg 212, 1213 XZ Hilversum, The Netherlands; 11 Department of Intensive Care Medicine, OLVG, Oosterpark 9, 1091 AC Amsterdam, The Netherlands

**Keywords:** Out-of-hospital cardiac arrest, Ischemia/reperfusion injury, Post-cardiac arrest syndrome, Cardiac arrest, Free radicals, Reactive oxygen species, Oxidative stress, Vitamin C, Ascorbic acid

## Abstract

**Background:**

High-dose intravenous vitamin C directly scavenges and decreases the production of harmful reactive oxygen species (ROS) generated during ischemia/reperfusion after a cardiac arrest. The aim of this study is to investigate whether short-term treatment with a supplementary or very high-dose intravenous vitamin C reduces organ failure in post-cardiac arrest patients.

**Methods:**

This is a double-blind, multi-center, randomized placebo-controlled trial conducted in 7 intensive care units (ICUs) in The Netherlands. A total of 270 patients with cardiac arrest and return of spontaneous circulation will be randomly assigned to three groups of 90 patients (1:1:1 ratio, stratified by site and age). Patients will intravenously receive a placebo, a supplementation dose of 3 g of vitamin C or a pharmacological dose of 10 g of vitamin C per day for 96 h. The primary endpoint is organ failure at 96 h as measured by the Resuscitation-Sequential Organ Failure Assessment (R-SOFA) score at 96 h minus the baseline score (delta R-SOFA). Secondary endpoints are a neurological outcome, mortality, length of ICU and hospital stay, myocardial injury, vasopressor support, lung injury score, ventilator-free days, renal function, ICU-acquired weakness, delirium, oxidative stress parameters, and plasma vitamin C concentrations.

**Discussion:**

Vitamin C supplementation is safe and preclinical studies have shown beneficial effects of high-dose IV vitamin C in cardiac arrest models. This is the first RCT to assess the clinical effect of intravenous vitamin C on organ dysfunction in critically ill patients after cardiac arrest.

**Trial registration:**

ClinicalTrials.gov NCT03509662. Registered on April 26, 2018. https://clinicaltrials.gov/ct2/show/NCT03509662European Clinical Trials Database (EudraCT): 2017-004318-25. Registered on June 8, 2018. https://www.clinicaltrialsregister.eu/ctr-search/trial/2017-004318-25/NL

**Supplementary Information:**

The online version contains supplementary material available at 10.1186/s13063-021-05483-3.

## Background

Intravenous treatment with high-dose vitamin C may improve the clinical outcome of post-cardiac arrest patients with ROSC (return of spontaneous circulation), because reactive oxygen species (ROS), generated during the systemic ischemic-reperfusion response, contribute to organ damage and death [1, 2]. Vitamin C directly scavenges free radicals, repairs oxidized scavengers such as glutathione, and reduces the production of ROS [3]. As a result, it may reduce ischemia/reperfusion injury. Due to the massive release of ROS, vitamin C stores become exhausted and vitamin C plasma concentrations decrease to deficiency levels within 3 days after a cardiac arrest [4–6]. Enteral supplementation of vitamin C cannot restore plasma concentrations in critically ill patients due to limited absorption and acutely increased requirements [7–10]. Intravenous supplementation is therefore required to restore the deficiency [11–14] or to achieve higher plasma concentrations [12].

Beneficial effects of high-dose intravenous (IV) vitamin C on myocardial and cerebral ischemia-reperfusion injury have been reported in preclinical studies [2]. In clinical studies, IV administration of vitamin C reduced cardiac injury during percutaneous coronary intervention (PCI) [15] and reduced the incidence of atrial fibrillation following cardiac surgery [16]. The effects of IV vitamin C after cardiac arrest have been demonstrated in preclinical studies, but not in patients [11], and the optimal dose has not been determined yet.

In septic patients, a population also exposed to severe oxidative stress, three small studies and one larger randomized controlled trial have shown promising results. High-dose intravenous vitamin C alone [17, 18], or in combination with thiamine and hydrocortisone [19, 20], improved organ failure recovery, shock reversal, and survival. Results on organ failure improvement and vasopressor requirement in other recently published trials are not uniform [21–28]. Ongoing randomized controlled trials hopefully provide more conclusive answers [29].

Currently, the prognosis of cardiac arrest patients remains poor [30]. The most important therapeutic target in out-of-hospital cardiac arrest (OHCA) is shortening the time to ROSC (minimizing no flow and low flow state) by early chest compressions and defibrillation. Once the circulation has recovered, no effective therapy that diminishes the ischemia/reperfusion injury has been developed yet besides targeted temperature management [1].

Therefore, we want to study whether a short-term treatment with supplementary or high-dose intravenous vitamin C, when administered in the early phase of post-cardiac arrest reperfusion, can limit organ damage.

## Methods

### Study design

The early high-dose vitamin C in post-cardiac arrest syndrome (VITaCCA) trial is a double-blind, multi-center, randomized placebo-controlled trial with a three-arm comparative design. The study was approved by the ethics committee at Amsterdam UMC, location VUmc (VU University Medical Centre Protocol Record METC-2018.120) and will be performed in adherence to the Declaration of Helsinki. The trial was prospectively registered at ClinicalTrials.gov on April 26, 2018, with identifier NCT03509662. The recruiting of patients started on October 7, 2019.

This clinical trial will be conducted in seven Dutch ICUs: Amsterdam UMC, Location VUmc (coordinating center), Gelderse Vallei Hospital, Franciscus Gasthuis & Vlietland, Tergooi Hospital, Amphia Hospital, Erasmus MC and OLVG, location East.

### Population

**Fig. 1:**
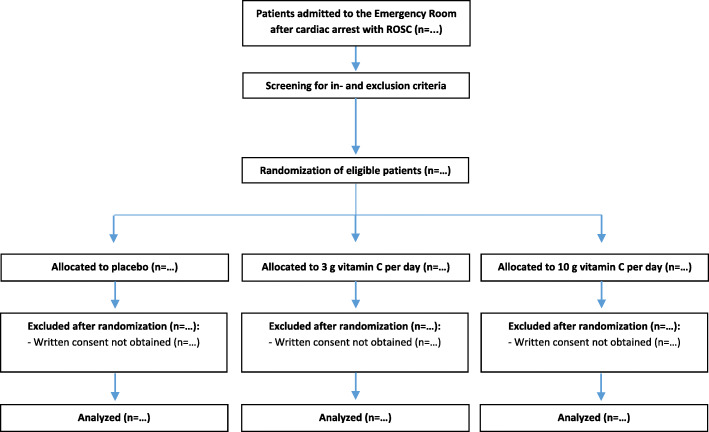
Flow chart of the study protocol

The study population consists of adult patients who suffered an out-of-hospital cardiac arrest. Patients must meet all of the inclusion criteria and none of the exclusion criteria in order to participate in this study (Table 1). Patients will be screened for eligibility by the attending physicians. A study flow chart is provided in Fig. 1.

**Table 1:** Inclusion and exclusion criteria

Inclusion criteria	Exclusion criteria
An out-of-hospital cardiac arrest with return of spontaneous circulation (ROSC); Ventricular fibrillation (VF) or ventricular tachycardia (VT) as first registered cardiac rhythm^a^; Glasgow Coma Scale (GCS) ≤8^b^.	Terminal renal insufficiency, i.e., receiving renal replacement therapy (RRT); Known glucose 6-phosphate dehydrogenase deficiency (risk of hemolysis); History of urolithiasis, oxalate nephropathy, or hemochromatosis; Treatment limitations^c^.

^a^If an automated external defibrillator advised to shock, then VF or VT was registered by the device.

^b^The last GCS-score before the start of sedatives will be used.

^c^The presence of treatment limitations will only be assessed at the moment of randomization.

### Study protocol

#### Interventions

As soon as possible after arrival at the Emergency Department, patients will be randomly allocated to one of three treatment groups. Group 1 will be treated with a placebo (0.9% NaCl), group 2 with a twice-daily intravenous bolus of 1.5 g vitamin C, and group 3 with a twice-daily intravenous bolus of 5 g vitamin C for 4 days or until ICU discharge. Ascorbic acid ampoules (100 mg/ml) from Centrafarm, Etten-Leur, the Netherlands, will be used. Placebo or vitamin C (diluted in 0.9% NaCl) is administered intravenously per 50 ml and is infused in 15 min, each 12 h. The first dose of the study medication is aimed to be administered within 5 h after ROSC. A total of eight infusions will be given in 4 days. In addition, all patients will receive intravenous thiamine 200 mg every 12 h for 4 days or until ICU discharge to limit the oxalate production [19].

Further post-cardiac arrest care, including targeted temperature management (TTM), is in accordance with international guidelines [31] and local procedures [32]. Standard intravenous vitamin C supplementation is allowed for dosages up to 200 mg a day. In case of clinical indication, higher dosages of thiamine are permitted. In case of irreversible coma, the procedure to withdraw life-sustaining treatment will take place according to Dutch guidelines [32].

### Randomization, treatment allocation, and blinding

Patients will be randomized with the use of a computer-generated randomization list. The randomization will be stratified by site and age (≤66 and >66 years) in randomized blocks of 6. The patient will be randomized, and thereby automatically allocated to a treatment group, by an unblinded nurse or pharmacy assistant not involved in the direct care of the included patient. The study medication will be prepared by this unblinded health care worker in an amber-colored syringe that will be connected to an orange infusion line. A blinded nurse involved in the direct care of the included patient will administer the prepared study medication to the patient. The patient, physicians, and researchers involved are blinded for the allocated treatment during the entire study period. Unblinding will only take place in case of a suspected unexpected serious adverse reaction (SUSAR). Then, the administration of study medication will be stopped immediately.

### Deferred consent

The ethics committee approved to start with study interventions before informed consent is obtained to avoid treatment delay. The legal representative will be informed and asked for informed consent (“deferred informed proxy consent”) by the investigator or the treating physician within 72 h after ICU admission. If informed consent cannot be obtained within 72 h, the patient will be excluded, and data will no longer be used. If the patient dies within 72 h after inclusion, prior to the informed consent procedure, the patient will not be excluded and data will be used [33]. The participant will be informed about participation in the study, if recovered. The patient is free to fill in a withdrawal form and can decide to remove the collected data and body material.

### Data collection and outcome measures

#### Data collection

**Fig. 2:**
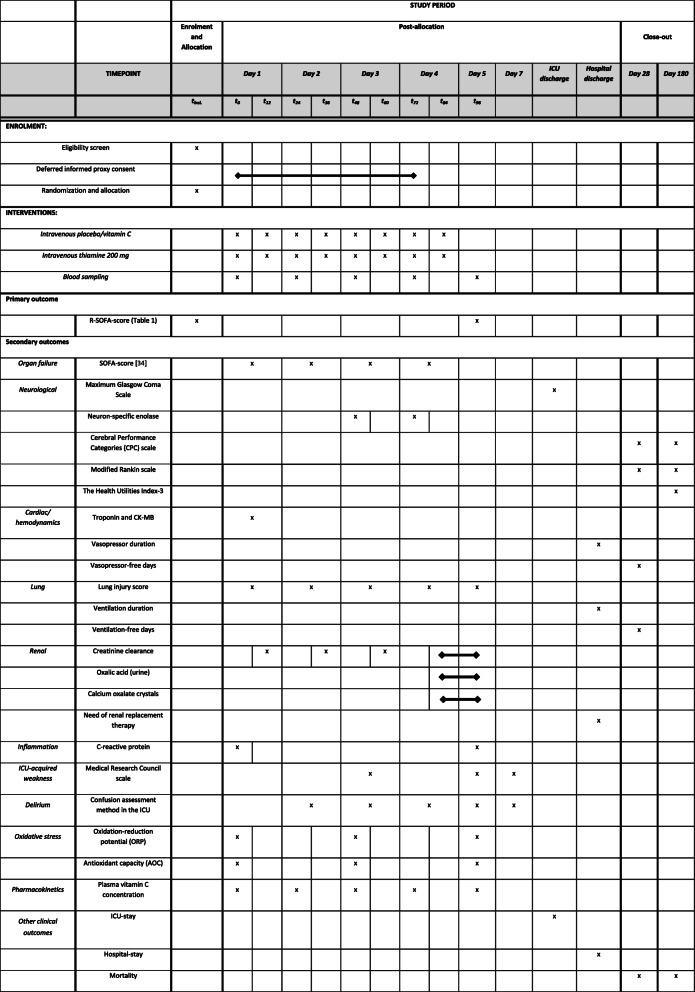
Data collection and follow-up of the participants in the VITaCCA-trial (SPIRIT figure)

Data collection time points are presented in Fig. 2 (SPIRIT Figure). T_0_ is the time point of the first dose, as *planned* in a written or electronic order. The actual moment of administering the first dose of study medication may be earlier or delayed due to logistical processes at the Emergency Department, but is aimed to take place within 5 h after ROSC. The next doses are every 12 h after T_0_. Therefore, the time difference between T_12_ and T_0_ can be less than 12 h.

To measure oxidative stress parameters and plasma vitamin C concentrations for pharmacokinetic modeling, the blood will be drawn daily in the coordinating center, before and directly after the infusion of study medication (baseline, trough, and peak plasma vitamin C concentrations). In other participating hospitals, if logistically feasible, the blood will be drawn daily before the infusion of study medication (baseline and trough plasma vitamin C concentrations).

Day 1 is defined as the first 24 h after T_0_. Each next 24 h time period is defined as the following day: day 2 is T_24_–T_48_, day 3 is T_48_–T_72_, and day 4 is T_72_–T_96_. Follow-up telephone interviews and data collection will take place around day 28 and day 180. After pseudonymization, all data will be stored in Castor EDC (Electronic Data Capture), eCRF.

#### Primary endpoint

The primary endpoint is the delta 96 h R-sequential organ failure assessment score (ΔR-SOFA_96-baseline_): The R-SOFA score at 96 h after randomization minus the score after ROSC (baseline R-SOFA score). ΔR-SOFA_96-baseline_ is, therefore, principally a rank-order endpoint. After correction for baseline organ failure, each patient can fare better, worse, or equally well compared to another patient. SOFA scores are calculated according to the NICE criteria [34]. However, the SOFA score used in the present study is an adjusted SOFA score, the so-called Resuscitation SOFA (R-SOFA), adjusted to assess the effect of the study intervention on organ dysfunction after cardiac arrest (see Additional File 1). In contrast to the classical SOFA score, for which worst values during a 24 h period are taken, the baseline and T_96_ SOFA scores are calculated within a shorter time window. The worst values during the first 24 h after ICU admission would not correctly reflect baseline organ dysfunction, as the study intervention which is administered early after ROSC could have influenced the degree of organ failure.

The adjustments are as follows:

1. Measurements must be reliable and should reflect the real organ function of the patient in a stable situation and not after an acute intervention.
  - Measurements should not be taken shortly after a bolus of catecholamines, propofol, or other sedatives, or after temporary ventilation with high oxygen fractions.
2. R-SOFA_baseline_: assessment should take place *prior* to the administration of the first study medication.
  - The first available bilirubin, platelet counts, and creatinine results will be taken, as these values do not change much during the first hours after hospital admission.
  - The last Glasgow Coma Scale (GCS) registered prior to the start of sedatives will be used.
  - Respiratory and/or hemodynamic parameters will be assessed in a time window of 2 h prior to the first dose. The worst validated parameters will be used.
    i. If no validated data are available, the first available validated data point after the first dose will be used. It is allowed to extend the time window with a maximum of 2 h after the first dose has been administered.
3. R-SOFA_96_: assessment should take place within a comparable time window of 2 h (T_95_–T_97_).
  - When bilirubin, platelet count and creatinine are not available within these 2 h, the nearest value within 24 h prior to T_96_, and the nearest value within 24 h after T_96_ will be averaged.
  - The last GCS without sedation prior to T_96_ will be assessed.
  - The worst validated respiratory and/or hemodynamic parameters will be used.
    i. If no validated data are available, the time window can be extended to 2 h before and 2 h after T_96_ (T_94_–T_98_).
  - If a recovered patient is discharged from the ICU within 96 h, the R-SOFA_96_ will be the R-SOFA score at ICU discharge.
  - Death at 96 h will be counted as the maximum R-SOFA score (24 points) as a worst-rank solution to informative censoring [35].
4. All SOFA components *must* be scored. If this is not possible based on the principles described above, these missing components will be discussed with an independent committee consisting of AdM, PE, and SR (coordinating investigators). They will decide which data point(s) can be used to score the organ(s), with our principles being the leading determinants.

#### Secondary endpoints

An overview of the secondary endpoints and data that will be collected is provided in Fig. 2 (SPIRIT Figure). An unadjusted SOFA-score [34] based on our 24 h time periods will be collected daily, as indicated in Fig. 2. The questionnaires will be carried out by telephone interviews [36]. In case a patient is not fully recovered and therefore not able to respond by phone, the questions will be asked to the legal representative of the patient.

Oxidative stress parameters will be measured using the RedoxSYS Diagnostic System (Aytu Bioscience, Englewood, CO, USA) [37]. In order to determine the plasma vitamin C concentration, blood samples will be processed immediately. After centrifugation, plasma is stabilized with 5.6% meta-phosphoric acid (1:5) to prevent oxidation of vitamin C and directly frozen at −80 °C until vitamin C measurement.

#### Other study parameters

Demographics of the patient will be collected, including age, sex, body-mass index (weight/height^2^), any relevant medical history, and medication use. Furthermore, prehospital data regarding the resuscitation will be obtained from the ambulance service (i.e., witnessed arrest, bystander CPR, AED usage, number of shocks provided, first monitored rhythm, drugs given, used airway device, and total duration of resuscitation). All personal information of potential and enrolled participants will be handled and stored confidentially, according to the Dutch law and regulations.

### Statistical methods

#### Primary outcome

The difference in ΔRSOFA_96-baseline_ score between intervention and control groups will be compared on a univariate basis (ANOVA with unadjusted analysis and after adjustment for, among others, age, site, prehospital parameters, and TTM) or (depending on normality) the Kruskal-Wallis test. In addition, both intervention groups will be combined and compared to placebo as well.

#### Secondary outcomes

Ordered categorical outcome variables (Glasgow Coma Score, Cerebral Performance Categories (CPC) scale, Modified Rankin scale (MRs), Health Utilities Index (HUI)) will be compared using the Cochran-Armitage test for trend. Continuous variables will be compared using linear mixed models or Friedman test as appropriate. Mortality will be analyzed using Kaplan-Meier curves and a Cox proportional hazards model with relevant prognostic covariates (especially time to return of spontaneous circulation). All statistical analyses will be performed according to the intention-to-treat principle. Moreover, the two vitamin C intervention groups will be combined for additional analyses.

#### Sample size calculation

ΔR-SOFA_96-baseline_  has an estimated standard deviation of 3.5 points (highest estimate based on previous data in our hospital and a recent systematic review [38]). The study is powered to detect a clinically meaningful effect of 2.0 SOFA points difference between the trial arms. Eighty-two patients per group are needed to detect a treatment effect of 2 SOFA points with 90% power and a two-sided alpha of 0.01. The type-I error rate is set at 0.01 instead of 0.05 to minimize the risk of false-positive results. The most important reason for reducing the false-positive risk beyond the 0.05 norm is that there is a large worldwide interest in high-dose vitamin C for critically ill patients. In this context, it was found important to reduce the fragility of the current trial in order to move beyond hypothesis-generating results. With an estimated 10% loss after randomization for late outcomes, the study aims to include 3×90 patients.

We consider a 2-point difference in ΔR-SOFA_96-baseline_ between the intervention and control groups a meaningful effect. Firstly, a 2-point difference is sufficiently large to be clinically relevant and to impact future practice. For example, 2 SOFA points are the difference between mild neurologic deficit and coma, between moderate kidney dysfunction and dialysis-dependent kidney failure, or between moderate oxygenation disturbance and life-threatening respiratory failure. Secondly, the association between ΔSOFA_96-baseline_ and outcomes is such that a 2-point difference is associated with measurable reductions in length-of-stay, mortality, and costs when the intervention is broadly implemented: each 1 point increase in ΔSOFA_96-baseline_ is associated with a 1.24 (95%CI 1.04–1.47) odds ratio of mortality [4, 39].

#### Safety

The theoretical risks of intravenous supplementation of high-dose vitamin C are a paradoxical pro-oxidative effect in case of iron overload, oxalate kidney stones, and factitious hyperglycemia. First, literature shows that in vivo, the antioxidant effect of vitamin C predominates this indirect pro-oxidative effect, even in iron-overloaded human plasma [40]. As a precaution, patients with known hemochromatosis will be excluded from this study. Second, calcium oxalate stones and oxalate nephrocalcinosis take months and years to develop, respectively, and no studies with short-term vitamin C administration reported kidney stone formation [17–23, 25–28, 41–48]. To limit the conversion of vitamin C to oxalate, all patients will receive thiamine 200 mg every 12 h for 4 days [19]. Third, high-dose IV vitamin C can lead to factitious hyperglycemia when measured with point-of-care devices [49], mostly at dosages much higher than used in our study. We will measure blood glucose during the period of vitamin C administration by blood gas analysis or in the central laboratory.

Case reports suggest that large dosages (>60 g) of IV vitamin C may induce hemolysis in patients with G6PD deficiency. However, at a low-moderate dose, vitamin C supplementation may instead be the recommended treatment of drug-induced G6PD deficiency and should therefore not be considered contraindicated [50]. Nonetheless, patients with known G6PD deficiency will be excluded in this present trial.

### Adverse events, serious adverse events, and suspected unexpected serious adverse reactions

Adverse events (“AEs”) will not be reported. Every 3 months the Principal Investigator shall send a line-listing of all serious adverse events (“SAEs”) to the Ethical Board of the coordinating center. For included patients, SAE’s will be collected up to 28 days after inclusion. For excluded patients, only the total time of participating in the study will be used. Suspected unexpected serious adverse reaction (“SUSAR”) will be reported immediately in accordance with the Dutch Medical Research Involving Human Subjects Act. Ancillary and post-trial care is not planned. An insurance has been taken out for everyone participating in this study. More insurance information can be found in the appendix of the information letter, which is available upon reasonable request.

### Data safety monitoring board and interim analysis

An independent data safety monitoring board (DSMB) consisting of three independent members (a biostatistician, an intensivist, and a cardiologist) will evaluate safety during the trial. A blinded interim analysis focused on mortality differences will be carried out by an independent methodologist after the inclusion of 135 evaluable patients. The DSMB will be blinded but may be unblinded by the independent methodologist on request without any further clarification. In case of significantly increased mortality in the vitamin C group(s) compared to placebo—and after a thorough examination of the clinical content—the DSMB can advise to stop the further recruitment. The DSMB will assess the unblinded data before arriving at a material recommendation.

### Monitoring

An independent monitor (quality officer) will monitor the study procedures and data quality according to the regulations described under Good Clinical Practice (GCP) in all participating centers. During onsite monitoring, the officer will perform source data verification of data in the Case Report Forms. In particular, adherence to inclusion and exclusion criteria, the deferred consent procedure, the primary endpoint, and the reporting of SAEs and SUSARs are subject to monitoring.

### Publication of trial results

The trial results will be reported in accordance with the Consolidated Standards of Reporting Trials [51].

### SPIRIT Checklist

The trial design follows the Standard Protocol Items Recommendations for Interventional Trials (SPIRIT). The SPIRIT Checklist is presented in Additional file 2.

## Discussion

VITaCCA is the first double-blind, multi-center, randomized placebo-controlled trial to compare the effects of early high-dose vitamin C on organ dysfunction in patients after cardiac arrest.

The optimal dosing for intravenous vitamin C in the context of critical illness is still unknown and may depend on the patients’ comorbid condition, on the severity of the acute disease, and on the subsequent degree of oxidative stress. We chose two different dose regimens in this study in order to observe differences in effect size between a supplementation and a pharmacological dose regimen. Our previous pharmacokinetic study demonstrates that 2 g a day was needed to restore plasma concentrations and that 10 g a day resulted in supranormal plasma concentrations [12]. This is in line with previous studies that show that 3 g a day was needed to restore deficient plasma concentrations [13]. Therefore, 3 g is chosen as a first dose regimen. To achieve supraphysiological plasma concentrations, 10 g will be used as a second dose regimen. This dose is in between 6 g and 16 g vitamin C a day, as used in previous studies [17–23, 25–28, 44–46]. Currently, the optimal length of vitamin C supplementation is also unknown. We chose for 4 days, which is in line with previous studies in sepsis [17–23, 25, 26, 44, 45]. After these 4 days, vitamin C administration will be lowered according to local guidelines, to allow the physiological signaling and repair function of low concentrations of ROS.

Delta SOFA score is chosen as the primary outcome since a recent meta-analysis demonstrates a strong reliable association with mortality, in comparison to a fixed day SOFA score [38]. Furthermore, a power calculation on a patient-oriented outcome such as mortality showed an unachievable required sample size of 3000 patients. The adjusted SOFA-score, the Resuscitation SOFA-score (R-SOFA), is designed to reliably assess baseline organ failure of post-cardiac arrest patients.

### Limitation

We intended to use ready-to-use study medication produced by a pharmacy, but during the storage period, many (unknown) degradation products were formed, possibly due to the air permeability of the polypropylene vials. Therefore, we opted for a fresh preparation of the study medication as described in this protocol. A separate study is being conducted to identify and quantify the degradation products in different vitamin C solutions.

In conclusion, this study investigates the effect of early high-dose vitamin C in post-cardiac arrest patients on organ dysfunction. If that proofs to be the case, intravenous vitamin C administration can be considered as a novel treatment for post-cardiac arrest patients.

### Trial status

The first patient was included on October 20, 2019. The current version of the study protocol is version 9, dated March 17, 2021. The estimated study completion date is January 2023.

## Supplementary Information

**Additional file 1:.** Supplementary Table 1. Resuscitation Sequential Organ Failure Assessment Score (R-SOFA)

**Additional file 2:.** SPIRIT 2013 Checklist: Recommended items to address in a clinical trial protocol and related documents

## Data Availability

The datasets will be available from the corresponding author on reasonable request.

## Abbreviations

AE: Adverse event
AED: Automatic external defibrillator
AOC: Antioxidant capacity
ANOVA: Analysis of variance
CPC: Cerebral performance categories
CPR: Cardiopulmonary resuscitation
DSMB: Data safety monitoring board
GCS: Glasgow coma scale
GCP: Good clinical practice
HUI: Health utilities index
ICU: Intensive care unit
IV: Intravenous
MRs: Modified ranking scale
NICE: Nationale Intensive Care Evaluatie
OHCA: Out-of-hospital cardiac arrest
ORP: Oxidation-reduction potential
PCI: Percutaneous coronary intervention
ROS: Reactive oxygen species
RRT: Renal replacement therapy
R-SOFA: Resuscitation-sequential organ failure assessment
ROSC: Return of spontaneous circulation
SAE: Serious adverse event
SUSAR: Suspected unexpected serious adverse reaction
TTM: Targeted temperature management
VF: Ventricular fibrillation
VT: Ventricular tachycardia
VITaCCA: Vitamin C in post-cardiac arrest syndrome
