# Supplementary material for: Early high-dose vitamin C in post-cardiac arrest syndrome (VITaCCA): study protocol for a randomized, double-blind, multi-center, placebo-controlled trial
Source: Trials. 2021 Aug 18;22:546. doi: 10.1186/s13063-021-05483-3 (PMC8371424; doi:10.1186/s13063-021-05483-3)
Supplement: Supplementary file 1 — Additional file 1:. Supplementary Table 1. Resuscitation Sequential Organ Failure Assessment Score (R-SOFA) [file 13063_2021_5483_MOESM1_ESM.docx]

| ***Supplementary Table 1.*** *Resuscitation Sequential Organ Failure Assessment Score (R-SOFA)* | | | | | |
| --- | --- | --- | --- | --- | --- |
|  | *Score* | | | | |
| *System* | *0* | *1* | *2* | *3* | *4* |
| ***Respiration*** | | | | | |
| *PaO_2_/FiO_2_, mmHg (kPa)^a^* | ≥*400 (53.3)* | *<400 (53.3)* | *<300 (40)* | *<200 (26.7) with respiratory support* | *<100 (13.3) with respiratory support*  *OR*  *special respiratory support* |
| ***Coagulation*** | | | | | |
| Platelets, x10^3^/µL | ≥150 | *<150* | *<100* | *<50* | *<20* |
| ***Liver*** | | | | | |
| *Bilirubin, mg/dL (µmol/L)* | *<1.2 (20)* | *1.2-1.9 (20-32)* | *2.0-5.9 (33-101)* | *6.0-11.9 (102-204)* | *>12.0 (204) OR  MARS* |
| ***Cardiovascular*** | | | | | |
|  | *MAP ≥70 mmHg* | *MAP <70 mmHg* | *Dopamine <5 mcg/kg/min  OR  dobutamine (any dose) OR PDI (any dose)* | *Dopamine 5.1-15 mcg/kg/min*  *OR*  *(nor)adrenaline ≤0.1 mcg/kg/min* | *Dopamine >15 mcg/kg/min*  *OR*  *(nor)adrenaline >0.1*  *OR*  *CAD* |
| ***Central nervous system*** | | | | | |
| *Glasgow Coma Scale score* | *15* | *13-14* | *10-12* | *6-9 OR ICP* | *<6* |
| ***Renal*** | | | | | |
| *Creatinine, mg/dL (µmol/L)* | *<1.2 (110)* | *1.2-1.9 (110-170)* | *2.0-3.4 (171-299)* | *3.5-4.9 (300-440)* | *>5.0 (440) OR*  *RRT* |
| *Abbreviations:*  *Special respiratory support: VV-ECLS, NO-ventilation, Double Lumen Ventilation or Partial Liquid Ventilation MARS: Molecular Adsorbent Recirculating System PDI: Phosphodiesterase Inhibitors*  *CAD: VA-ECLS, IABP, Impella ICP: Intracranial Pressure monitoring*  *RRT: Renal Replacement Therapy*  *^a^ If a patient is ventilated (also NIV or CPAP) and non-ventilated in the time window concerned, only the ventilated blood gas levels are used (when available).* | | | | | |
